# Supplementary material for: Tyrosine phosphorylation controlled poly(A) polymerase I activity regulates general stress response in bacteria
Source: Life Sci Alliance. 2022 Dec 19;6(3):e202101148. doi: 10.26508/lsa.202101148 (PMC9764084; doi:10.26508/lsa.202101148)
Supplement: Supplementary file 2 [file LSA-2021-01148_TableS2.docx]

**Table S2: List of PAPI target stress related genes and different stress responses**

| **Gene** | **Stress Response** | **Gene** | **Stress Response** | **Gene** | **Stress Response** |
| --- | --- | --- | --- | --- | --- |
| *aidB* | DNA damage | *glmY* | Acid Shock | *sugE* | Multiple Stresses |
| *aldB* | DNA damage | *glsA* | Acid Shock | *tnaA* | DNA damage |
| *alkA* | DNA damage | *hchA* | Starvation | *tnaB* | DNA damage |
| *bfr* | Oxidative Stress | *hdeA* | Acid Shock | *tqsA* | Biofilm Formers |
| *blc* | Oxidative Stress | *hdeB* | Acid Shock | *treA* | Cold Shock |
| *bolA* | Biofilm Formers | *hdeD* | Acid Shock | *umuD* | DNA damage |
| *bsmA* | Oxidative Stress | *ibpB* | Heat Shock | *uspA* | Multiple Stresses |
| *bssR* | Biofilm Formers | *iraD* | DNA damage | *uspB* | Multiple Stresses |
| *caiA* | Osmotic Shock | *katE* | Oxidative Stress | *uspC* | Osmotic Shock |
| *caiC* | Osmotic Shock | *ldcC* | Acid Shock | *uspD* | Osmotic Shock |
| *caiD* | Osmotic Shock | *loiP* | Heat Shock | *uspE* | Osmotic Shock |
| *caiE* | Osmotic Shock | *lsrA* | Multiple Stresses | *uspF* | Oxidative Stress |
| *caiF* | Osmotic Shock | *lsrB* | Multiple Stresses | *wrbA* | Oxidative Stress |
| *caiT* | Osmotic Shock | *lsrC* | Multiple Stresses | *yafP* | DNA damage |
| *cbpA* | DNA damage | *lsrF* | Multiple Stresses | *yafY* | Heat Shock |
| *clpA* | Heat Shock | *lsrG* | Multiple Stresses | *yagU* | Acid Shock |
| *clpB* | Heat Shock | *lsrK* | Multiple Stresses | *yahD* | Radiation |
| *clsB* | Osmotic Shock | *mrr* | DNA damage | *yaiA* | Oxidative Stress |
| *crl-1* | Heat Shock | *msrA* | Oxidative Stress | *ybdK* | Oxidative Stress |
| *cspD* | Cold Shock | *msyB* | Heat Shock | *ybiO* | Osmotic Shock |
| *cysD* | Oxidative Stress | *nhaA* | Acid Shock | *ycgB* | DNA damage |
| *cysN* | Oxidative Stress | *nlpE* | Envelope stress | *ydeI* | Oxidative Stress |
| *cysP* | Oxidative Stress | *osmC* | Oxidative Stress | *ydeM* | Biofilm Formers |
| *cysQ* | Oxidative Stress | *osmE* | Osmotic Shock | *ydiZ* | DNA damage |
| *cysW* | Oxidative Stress | *osmF* | Osmotic Shock | *yeaG* | Starvation |
| *deoA* | DNA damage | *osmY* | Osmotic Shock | *yeaH* | Starvation |
| *deoB* | DNA damage | *otsA* | Cold Shock | *yecS* | Oxidative Stress |
| *deoC* | DNA damage | *otsB* | Cold Shock | *yedZ* | Oxidative Stress |
| *dhaM* | DNA damage | *otsB* | Osmotic Shock | *yehW* | Osmotic Shock |
| *dinB* | DNA damage | *pgaA* | Biofilm Formers | *yehX* | Osmotic Shock |
| *dnaK* | Heat Shock | *phr* | Radiation | *yfcG* | Oxidative Stress |
| *dps* | Starvation | *pphA* | Multiple Stresses | *yfdK* | Oxidative Stress |
| *dsdA* | DNA damage | *psiE* | Starvation | *yfdY* | Biofilm Formers |
| *dsdX* | DNA damage | *raiA* | Cold Shock | *yfgG* | Heavy Metal stress |
| *elaB* | Multiple Stresses | *rclA* | Oxidative Stress | *ygaM* | Oxidative Stress |
| *entH* | Oxidative Stress | *rclR* | Oxidative Stress | *ygaU* | Osmotic Shock |
| *exoX* | DNA damage | *rmf* | Starvation | *yggE* | Oxidative Stress |
| *fliY* | Oxidative Stress | *sbmC* | DNA damage | *yghU* | Oxidative Stress |
| *ftnB* | Oxidative Stress | *slp* | Starvation | *ygiD* | Biofilm Formers |
| *gadA* | Acid Shock | *sufA* | Oxidative Stress | *ygiV* | Heavy Metal stress |
| *gadB* | Acid Shock | *sufB* | Oxidative Stress | *ygiW* | Oxidative Stress |
| *gadC* | Acid Shock | *sufC* | Oxidative Stress | *yhiM* | Acid Shock |
| *gadW* | Acid Shock | *sufD* | Oxidative Stress | *yibF* | Oxidative Stress |
| *gadX* | Acid Shock | *sufE* | Oxidative Stress | *yibH* | DNA damage |
| *glcF* | Oxidative Stress | *sufS* | Oxidative Stress | *yjaB* | Multiple Stresses |
