# Supplementary material for: Tyrosine phosphorylation controlled poly(A) polymerase I activity regulates general stress response in bacteria
Source: Life Sci Alliance. 2022 Dec 19;6(3):e202101148. doi: 10.26508/lsa.202101148 (PMC9764084; doi:10.26508/lsa.202101148)
Supplement: Supplementary file 3 [file LSA-2021-01148_Supplemental_Data_1.docx]

**Supplementary data**

**Tyrosine phosphorylation controlled poly(A) polymerase I activity regulates general stress response in bacteria**

**Supplementary Figures and Legends**

**Figure S1**: (A) Heat map showing gene expression profile of *pcnB*-null mutant (MG-*pcnB*) compared to wild type MG1655 cells as in Figure 1A-B. (B) List of select genes involved in various stress response pathways from the up-regulated genes on *pcnB*-null mutation. Important global regulators of stress response that are not affected by *pcnB*-null mutation is indicated below. (C) Viable colony counting from 10^-4^ diluted cells from stress treated and untreated cultures of MG1655, MG-*pcnB* and MG1655 transformed with pFLAG^B^-*pcnB* plasmid as in Figure 1E on LB media expressed as colony forming unit (CFU/mL x 10^5^) cells.

**Figure S2:** (A) A schematic of 3′-RACE assay. Engineered oligo-dT primer with a unique sequence at the 5ʹ-end, positions of gene specific forward primer (GSP) and adapter reverse primer (AUAP) are indicated. (B) qRT-PCR analysis of *pcnB* mRNA level from MG1655 cells in the presence and absence of treatment with different stressors as indicated (p-value <0.01 under all conditions). (C) In vitro polyadenylation assay of recombinant His-PAPI and mammalian canonical PAPα as in Figure 2F. (D) In vitro polyadenylation assay of His-PAPI after in vitro phosphorylation with stress primed (NaCl and MNNG) and unprimed cell lysates. (E) In vitro polyadenylation assay of MG1655 cell lysates as in Figure 2F after treatment with oligo-dT and RNaseH as indicated. (F-G) Quantification of relative intensity of in vitro polyadenylation assays in D and E respectively by Image J software. Error bar represents SEM of relative intensities from n=3 independent gels. (H) qRT-PCR analysis of stress response genes involved in different stresses assayed after treatment with NaCl stress. Error bar represents SEM of n=3 independent experiments (p values <0.001 for *bsmA, cspD*, *rmf*, <0.002 for *clpA, uspA*, <0.02 for *dsdA,* <0.02 for *aldB*).

**Figure S3:** (A) Western blot analysis using phosphotyrosine (P^Tyr^) and phosphoserine (P^Ser^) specific antibodies as in Figure 3A from recombinant His-PAP purified from BL21(DE3) cells. Control blot of His-PAPI using specific antibody against hexa-histidine tag (His) is shown below. (B) Quantification of relative intensities of each band of the blot shown in Figure 3C. Error bar represents SEM (n=3 independent blotting experiments) (C) Western blot analysis of His-PAPI purified from BL21(DE3) cells after treatment with multiple stresses with phosphotyrosine (P^Tyr^) specific antibody as indicated. Control His-PAPI blot is shown below. (D) Quantification of relative intensity of phosphotyrosine level after treatment with different stresses from C. (E) In vitro polyadenylation assay of recombinant His-PAPI protein after in vitro phosphorylation with stress (NaCl) treated and untreated MG1655 cell lysates as indicated. (F) Quantification of relative intensity of in vitro polyadenylation assays in E. Error bar represents SEM of relative intensities of 3 independent gels. (G) Western blot analysis using phosphotyrosine (P^Tyr^) of FLAG PAPI after in vitro phosphorylation with untreated or stress (NaCl) treated cell lysate. Dephosphorylation with either λ phosphatase (λppt) or bacterial tyrosine-protein phosphatase (YopH) in the presence or absence of phosphatase inhibitor, sodium vanadate (Inh) is indicated. (H) Quantification of relative intensity of in vitro polyadenylation assays shown in Figure 3I. Error bar represents SEM of relative intensities of 3 independent experiments (I) In silico analysis of putative tyrosine phosphorylation sites in PAPI using NetPhos 3.1. Score threshold was set at 0.5 to select the putative sites as per the software instructions. 5 putative tyrosine sites above the threshold are indicated by arrow. (J) Western blot analysis for MG1655 cells treated with or without stress after peptide competition assay for P^Y202^ antibody with phosphopeptide (P^Y202^) or non-phospho peptide (NP). Control FLAG blots are shown

**Figure S4:** (A) In vitro polyadenylation assay of immunopurified wild type FLAG-PAPI and various phospho-mutants (Y202F, Y341F) in the presence of absence of stress (NaCl) treatment. (B) Quantification of relative intensity of in vitro polyadenylation assays in “A”. Error bar represents SEM. (C) Viable colony counting of various mutants in pFLAG^B^-*pcnB* [Y60F, Y169F, Y170F, Y202F, Y341F] and wild type construct (WT) expressed in MG-*pcnB* cells in the presence of NaCl stress treatment represented in CFU/mL (D) Growth curve analysis of various mutants in pFLAG^B^-*pcnB* [Y60F, Y169F, Y170F, Y202F, Y341F] and wild type construct (WT) expressed in MG-*pcnB* cells in the presence of NaCl stress treatment. (E) Comparison of interaction between Y202 and T227 residues in the wild type PAP I (top panel) and in the PAPI-P^Y202^ (bottom panel). (F) Analysis of number of hydrogen bonds formed in wild type PAPI (top panel) and in the PAPI-P^Y202^ (bottom panel) in the 300 ns simulation time frame.

**Figure S5**: (A) In vitro kinase assay of radioactive ^32^P-ATP incorporation in His-PAPI using stress-primed cell lysates of various strains as indicated (B) In vitro kinase assay of radioactive ^32^P-ATP incorporation in various PAPI mutants (Y60F, Y169F ,Y170F, Y202F, and Y341F) of His-PAPI protein using purified Wzc protein kinase. (C) Dilution spotting of MG1655, *bipA-*null mutant (MG-*bipA*), *etk*-null mutant (MG-*etk*), *wzc*-null mutant (MG-*wzc*), and *ydiB*-null mutant (MG-*ydiB*) strains spotted at a dilution from 10^-2^ to 10^-6^ after treatment with or without NaCl. (D) Schematics of *wzc* promoter sequence showing RpoD- and RpoE- dependent promoter regions upstream of *wzc* transcription unit along with their respective transcription start sites. (E) Western blot analysis for Wzc tyrosine autophosphorylation using ectopically expressed FLAG-Wzc with or without stress treatment in the cell as indicated. Control PAPI-P^Y202^ and FLAG blot is also shown. (F) Schematics of two different *osmY* reporter constructs; pFLAG^B^-*osmY*^NS-P^ (expressing a FLAG epitope tagged OsmY with *osmY* 3′-UTR but driven from a stress insensitive promoter) and pFLAG^B^-*osmY*^SS-P^ (expressing a FLAG epitope tagged OsmY with *osmY* 3′-UTR and driven from stress sensitive *osmY* promoter). Promoter sequence and positions of *osmY* cds and UTR regions are indicated. (G) Sequence chromatogram of *osmY* mRNA UTR showing PA-tail addition and the region where PA-tail is added after transcription termination. (H) qRT-PCR analysis of stress response genes *osmY* and *uspE* assayed after treatment with NaCl in various strains as indicated. Error bar represents SEM of n=3 independent experiments (p values <0.001 for *osmY* and *uspE* gene under all conditions). (I) Viable colony counting from 10^-4^ diluted cells from stress treated cultures of MG1655, MG-*rpoS*, MG-*rpoS*-*pcnB*, MG-*rpoS-wzc* strains expressed as colony forming unit (CFU/mL) cells at two different time points post stress treatment as indicated. Error bar represents SEM of n=3 independent cultures.

**Figure S6**: A model depicting how Y202 PAP phosphorylation mediated by bacterial tyrosine kinase Wzc regulates general stress response in bacteria.

**List of antibodies**

# Rabbit monoclonal anti-FLAG (Sigma) antibody, mouse monoclonal anti-FLAG M2 (Sigma) antibody, mouse monoclonal anti-phospho-tyrosine (PY 99) (Santacruz) antibody, mouse monoclonal anti-phospho-serine (16B4) (Santacruz) antibody, mouse monoclonal anti-neomycin phospho transferase II antibody (NTP) (abcam) antibody, mouse monoclonal anti-His tag (Immunotag) antibody, rabbit polyclonal anti-PAPI antibody (in-house, abgenex), rabbit polyclonal anti-P^Y202^ antibody (in-house, abgenex) were used for Western blot or IP experiments.

# List of peptides

# Phosphopeptide : IRLIGNPETRY(p)REDPVRMLR

# Nonphospho peptide : IRLIGNPETRYREDPVRMLR

**Table S2: List of PAPI target stress related genes and different stress responses**

| **Gene** | **Stress Response** | **Gene** | **Stress Response** | **Gene** | **Stress Response** |
| --- | --- | --- | --- | --- | --- |
| *aidB* | DNA damage | *glmY* | Acid Shock | *sugE* | Multiple Stresses |
| *aldB* | DNA damage | *glsA* | Acid Shock | *tnaA* | DNA damage |
| *alkA* | DNA damage | *hchA* | Starvation | *tnaB* | DNA damage |
| *bfr* | Oxidative Stress | *hdeA* | Acid Shock | *tqsA* | Biofilm Formers |
| *blc* | Oxidative Stress | *hdeB* | Acid Shock | *treA* | Cold Shock |
| *bolA* | Biofilm Formers | *hdeD* | Acid Shock | *umuD* | DNA damage |
| *bsmA* | Oxidative Stress | *ibpB* | Heat Shock | *uspA* | Multiple Stresses |
| *bssR* | Biofilm Formers | *iraD* | DNA damage | *uspB* | Multiple Stresses |
| *caiA* | Osmotic Shock | *katE* | Oxidative Stress | *uspC* | Osmotic Shock |
| *caiC* | Osmotic Shock | *ldcC* | Acid Shock | *uspD* | Osmotic Shock |
| *caiD* | Osmotic Shock | *loiP* | Heat Shock | *uspE* | Osmotic Shock |
| *caiE* | Osmotic Shock | *lsrA* | Multiple Stresses | *uspF* | Oxidative Stress |
| *caiF* | Osmotic Shock | *lsrB* | Multiple Stresses | *wrbA* | Oxidative Stress |
| *caiT* | Osmotic Shock | *lsrC* | Multiple Stresses | *yafP* | DNA damage |
| *cbpA* | DNA damage | *lsrF* | Multiple Stresses | *yafY* | Heat Shock |
| *clpA* | Heat Shock | *lsrG* | Multiple Stresses | *yagU* | Acid Shock |
| *clpB* | Heat Shock | *lsrK* | Multiple Stresses | *yahD* | Radiation |
| *clsB* | Osmotic Shock | *mrr* | DNA damage | *yaiA* | Oxidative Stress |
| *crl-1* | Heat Shock | *msrA* | Oxidative Stress | *ybdK* | Oxidative Stress |
| *cspD* | Cold Shock | *msyB* | Heat Shock | *ybiO* | Osmotic Shock |
| *cysD* | Oxidative Stress | *nhaA* | Acid Shock | *ycgB* | DNA damage |
| *cysN* | Oxidative Stress | *nlpE* | Envelope stress | *ydeI* | Oxidative Stress |
| *cysP* | Oxidative Stress | *osmC* | Oxidative Stress | *ydeM* | Biofilm Formers |
| *cysQ* | Oxidative Stress | *osmE* | Osmotic Shock | *ydiZ* | DNA damage |
| *cysW* | Oxidative Stress | *osmF* | Osmotic Shock | *yeaG* | Starvation |
| *deoA* | DNA damage | *osmY* | Osmotic Shock | *yeaH* | Starvation |
| *deoB* | DNA damage | *otsA* | Cold Shock | *yecS* | Oxidative Stress |
| *deoC* | DNA damage | *otsB* | Cold Shock | *yedZ* | Oxidative Stress |
| *dhaM* | DNA damage | *otsB* | Osmotic Shock | *yehW* | Osmotic Shock |
| *dinB* | DNA damage | *pgaA* | Biofilm Formers | *yehX* | Osmotic Shock |
| *dnaK* | Heat Shock | *phr* | Radiation | *yfcG* | Oxidative Stress |
| *dps* | Starvation | *pphA* | Multiple Stresses | *yfdK* | Oxidative Stress |
| *dsdA* | DNA damage | *psiE* | Starvation | *yfdY* | Biofilm Formers |
| *dsdX* | DNA damage | *raiA* | Cold Shock | *yfgG* | Heavy Metal stress |
| *elaB* | Multiple Stresses | *rclA* | Oxidative Stress | *ygaM* | Oxidative Stress |
| *entH* | Oxidative Stress | *rclR* | Oxidative Stress | *ygaU* | Osmotic Shock |
| *exoX* | DNA damage | *rmf* | Starvation | *yggE* | Oxidative Stress |
| *fliY* | Oxidative Stress | *sbmC* | DNA damage | *yghU* | Oxidative Stress |
| *ftnB* | Oxidative Stress | *slp* | Starvation | *ygiD* | Biofilm Formers |
| *gadA* | Acid Shock | *sufA* | Oxidative Stress | *ygiV* | Heavy Metal stress |
| *gadB* | Acid Shock | *sufB* | Oxidative Stress | *ygiW* | Oxidative Stress |
| *gadC* | Acid Shock | *sufC* | Oxidative Stress | *yhiM* | Acid Shock |
| *gadW* | Acid Shock | *sufD* | Oxidative Stress | *yibF* | Oxidative Stress |
| *gadX* | Acid Shock | *sufE* | Oxidative Stress | *yibH* | DNA damage |
| *glcF* | Oxidative Stress | *sufS* | Oxidative Stress | *yjaB* | Multiple Stresses |

**Table S3: List of primers used in the study**

| **qRT-PCR analysis** | | | |
| --- | --- | --- | --- |
| Gene | Forward (5ʹ to 3ʹ direction) | | Reverse (5ʹ to 3ʹ direction) |
| *aidB* | GCAAACTCACACCGTTTTTAATC | | ATAATTCAGGCGGATTCACATT |
| *aldB* | GGTGGGATCAGTGAAGTTGATAG | | TTTCCATTAGCAGCAGTACAGAA  TTTCCATTAGCAGCAGTACAGAA |
| *bsmA* | TGGTTAGCAGGAAACGTAATAGC | | GCTTACGCTACCTATTCGCTGTA |
| *clpA* | CTTATCCACCAGGATAACAGCAC | | CTTCCAGAGAAACACCTTTCTGA |
| *cspD* | GTGGTTCAACAATGCCAAAG | | TACTTCGACGGGCACAATAA |
| *cspE* | GTTTCATTACTCCGGAAGACG | | CTTTGGCACCGTTAGTGATTT |
| *deoA* | CTCTATTCTGGCGAAGAAACTTG | | AGTACCTGATTCATGTCGGTGA |
| *dsdA* | GGCTGAAGGTTTACCTTATGTTG | | TGATCGGTTGCTGATATTCTTTT |
| *dxs* | TTCATCAAGCGGTTTCACA | | CGAGAAACTGGCGATCCTTA |
| *lpp* | GCTCCAGCAACGCTAAAATC | | ATGTTGTCCAGACGCTGGTT |
| *otsA* | GTACGTTTTTCAGTTCCGCTTTA | | TCCTGGATTGTCTTTCTAACCTG |
| *osmY* | AAAACTCTGCTGGCTGTAATGTT | | GCTCTTGATGTTGTCATGATCC |
| *rmf* | AAAACGAGATCGCCTGGA | | AGCCATTGTGACCTTTGATTC |
| *RNAI* | ATTTGGTATCTGCGCTCTG | | GTTTGTTTGCCGGATCAA |
| *rpsO* | CGTTTCTGAGTTTGGTCGTG | | TTTTGTGCTCTGCAAAGTGG |
| *secG* | AGGTAAAGGCGCTGATATGG | | TTGATGTTACCCAGCACCAG |
| *trpA* | TATGCCCAGTGCGAAAAAGT | | TGCTCGTGACAGCAAATAGG |
| *uspA* | GCTTTCCACTAATGCAGGCTA | | GCGGAACAATCAGCATATCA |
| *uspC* | CCGGAAATGTACAATCAATTAGC | | ATCGAAATGATGCTTGTGACATA |
| *uspE* | AGTCAACCATACCGAAGTTCATC | | CTCTTCTGGCAGACCTTTTTCTA |
| *wrbA* | GGCGCTGAAGTTGTCGTTAA | | ATTTGACCGGACATGTTGCC |
| *wzc* | TCGTTGTTTAATCGCGGCAT | | CTACGGATGGCTTCAATCGC |
| **3ʹ- RACE Assay** | | | |
| *osmY* FP | | CTCCGGTACCGTCGATTCT | |
| Adapter primer | | GGCCACGCGTCGACTAGTACTTTTTTTTTTTTTTTTT | |
| AUAP RP | | GGCCACGCGTCGACTAGTAC | |
| **Site Directed Mutagenesis** | | | |
| *pcnB*-Y60F | | FP: CCCTGAAGGTAATGTTCAGGCTCAATAAAGC | |
|  |  | RP: GCTTTATTGAGCCTGAACATTACCTTCAGGG | |
| *pcnB*-Y169,170F | | FP: CTATCAACAGCCTGTTTTTCAGCGTAGCGGATT | |
|  |  | RP: AATCCGCTACGCTGAAAAACAGGCTGTTGATAG | |
| *pcnB*-Y202F | | FP: CCGGAAACGCGCTTCCGTGAAGATCCG | |
|  |  | RP: CGGATCTTCACGGAAGCGCGTTTCCGG | |
| *pcnB-*Y341F | | FP: AAGCGGCCTGACCTTTCACGACGCTTTCG | |
|  |  | RP: CGAAAGCGTCGTGAAAGGTCAGGCCGCTT | |
| *wzc-*K540R | | FP:CCCGTCAATTGGTAGAACCTTTGTCTGCG | |
|  |  | RP:CGCAGACAAAGGTTCTACCAATTGACGGG | |
| **Primers for p-FLAG^B^ construction** | | | |
| -35 Sequence | | FP:AAATTAACCCACTAAATTGACAAAAAGCTGGAGCT | |
|  |  | RP:AGCTCCAGCTTTTTGTCAATTTAGTGGGTTAATTT | |
| -10 Sequence | | FP: CTGGAGCTCCACTATATTGGCGGCCGCC | |
|  |  | RP: GGCGGCCGCCAATATAGTGGAGCTCCAG | |
| Shine Dalgarno Sequence | | FP: CTATATTGGCGGCCAGAAGGAGCCACCATGGATTA | |
|  |  | RP: TAATCCATGGTGGCTCCTTCTGGCCGCCAATATAG | |
| Termination site | | FP: TGAATGGCGAATGGCGAGCCAATTTTTAAGTGTAT | |
|  |  | RP: ATACACTTAAAAATTGGCTCGCCATTCGCCATTCA | |
| -35 Sequence for pFLAG^B^-*osmY^SS-P^* | | FP: AAATTAACCCTCACTATCCCGAGCGAAAAGCTGGAGCTC | |
|  |  | RP: GAGCTCCAGCTTTTCGCTCGGGATAGTGAGGGTTAATTT | |
| Transcription Startsite for pFLAG^B^-*osmY^SS-P^* | | FP: GAGCTCCACTATATTTAACAAAAAGAAGGAGCCACCA | |
|  |  | RP: TGGTGGCTCCTTCTTTTTGTTAAATATAGTGGAGCTC | |
| **Sequencing Primer** | | | |
| T7 promoter | | TAATACGACTCACTATAG | |
| **Primer for Cloning** | | | |
| pFLAG^B^-*pcnB* | | FP: CGCGGATCCGATTTTTACCCGAGTCGCT | |
|  |  | RP: CCGGAATTCCGTCATGCGGTACCCTCAC | |
| pFLAG^B^-*osmY* | | FP : CGGGATCCGATGACTATGACAAGACTGAAGA | |
|  |  | RP: GAATTCAAGCGTCTCCTTTACCATAGT | |
| pFLAG^B^-*wzc* | | FP: CGCGGATCCGATGACAGAAAAAGTAAAACAAC | |
|  |  | RP: GAATTCTTTCGCATCCGACTTATATTCG | |
| pET-*pcnB* | | FP: CCATGCCATGGGCATTTTTACCCGAGTCGCTAA | |
|  |  | RP: CCGCTCGAGCGGTGCGGTACCCTCACGACGT | |
| **RNA Oligos** | | | |
| A_45_ oligo | | AGGGAUAGGGAUAGGGAUUAGGGAUAGGGAAAAAAAAA | |
