# Supplementary material for: Tyrosine phosphorylation controlled poly(A) polymerase I activity regulates general stress response in bacteria
Source: Life Sci Alliance. 2022 Dec 19;6(3):e202101148. doi: 10.26508/lsa.202101148 (PMC9764084; doi:10.26508/lsa.202101148)
Supplement: Supplementary file 4 [file LSA-2021-01148_TableS3.docx]

**Table S3: List of primers used in the study**

| **qRT-PCR analysis** | | | |
| --- | --- | --- | --- |
| Gene | Forward (5ʹ to 3ʹ direction) | | Reverse (5ʹ to 3ʹ direction) |
| *aidB* | GCAAACTCACACCGTTTTTAATC | | ATAATTCAGGCGGATTCACATT |
| *aldB* | GGTGGGATCAGTGAAGTTGATAG | | TTTCCATTAGCAGCAGTACAGAA  TTTCCATTAGCAGCAGTACAGAA |
| *bsmA* | TGGTTAGCAGGAAACGTAATAGC | | GCTTACGCTACCTATTCGCTGTA |
| *clpA* | CTTATCCACCAGGATAACAGCAC | | CTTCCAGAGAAACACCTTTCTGA |
| *cspD* | GTGGTTCAACAATGCCAAAG | | TACTTCGACGGGCACAATAA |
| *cspE* | GTTTCATTACTCCGGAAGACG | | CTTTGGCACCGTTAGTGATTT |
| *deoA* | CTCTATTCTGGCGAAGAAACTTG | | AGTACCTGATTCATGTCGGTGA |
| *dsdA* | GGCTGAAGGTTTACCTTATGTTG | | TGATCGGTTGCTGATATTCTTTT |
| *dxs* | TTCATCAAGCGGTTTCACA | | CGAGAAACTGGCGATCCTTA |
| *lpp* | GCTCCAGCAACGCTAAAATC | | ATGTTGTCCAGACGCTGGTT |
| *otsA* | GTACGTTTTTCAGTTCCGCTTTA | | TCCTGGATTGTCTTTCTAACCTG |
| *osmY* | AAAACTCTGCTGGCTGTAATGTT | | GCTCTTGATGTTGTCATGATCC |
| *rmf* | AAAACGAGATCGCCTGGA | | AGCCATTGTGACCTTTGATTC |
| *RNAI* | ATTTGGTATCTGCGCTCTG | | GTTTGTTTGCCGGATCAA |
| *rpsO* | CGTTTCTGAGTTTGGTCGTG | | TTTTGTGCTCTGCAAAGTGG |
| *secG* | AGGTAAAGGCGCTGATATGG | | TTGATGTTACCCAGCACCAG |
| *trpA* | TATGCCCAGTGCGAAAAAGT | | TGCTCGTGACAGCAAATAGG |
| *uspA* | GCTTTCCACTAATGCAGGCTA | | GCGGAACAATCAGCATATCA |
| *uspC* | CCGGAAATGTACAATCAATTAGC | | ATCGAAATGATGCTTGTGACATA |
| *uspE* | AGTCAACCATACCGAAGTTCATC | | CTCTTCTGGCAGACCTTTTTCTA |
| *wrbA* | GGCGCTGAAGTTGTCGTTAA | | ATTTGACCGGACATGTTGCC |
| *wzc* | TCGTTGTTTAATCGCGGCAT | | CTACGGATGGCTTCAATCGC |
| **3ʹ- RACE Assay** | | | |
| *osmY* FP | | CTCCGGTACCGTCGATTCT | |
| Adapter primer | | GGCCACGCGTCGACTAGTACTTTTTTTTTTTTTTTTT | |
| AUAP RP | | GGCCACGCGTCGACTAGTAC | |
| **Site Directed Mutagenesis** | | | |
| *pcnB*-Y60F | | FP: CCCTGAAGGTAATGTTCAGGCTCAATAAAGC | |
|  |  | RP: GCTTTATTGAGCCTGAACATTACCTTCAGGG | |
| *pcnB*-Y169,170F | | FP: CTATCAACAGCCTGTTTTTCAGCGTAGCGGATT | |
|  |  | RP: AATCCGCTACGCTGAAAAACAGGCTGTTGATAG | |
| *pcnB*-Y202F | | FP: CCGGAAACGCGCTTCCGTGAAGATCCG | |
|  |  | RP: CGGATCTTCACGGAAGCGCGTTTCCGG | |
| *pcnB-*Y341F | | FP: AAGCGGCCTGACCTTTCACGACGCTTTCG | |
|  |  | RP: CGAAAGCGTCGTGAAAGGTCAGGCCGCTT | |
| *wzc-*K540R | | FP:CCCGTCAATTGGTAGAACCTTTGTCTGCG | |
|  |  | RP:CGCAGACAAAGGTTCTACCAATTGACGGG | |
| **Primers for p-FLAG^B^ construction** | | | |
| -35 Sequence | | FP:AAATTAACCCACTAAATTGACAAAAAGCTGGAGCT | |
|  |  | RP:AGCTCCAGCTTTTTGTCAATTTAGTGGGTTAATTT | |
| -10 Sequence | | FP: CTGGAGCTCCACTATATTGGCGGCCGCC | |
|  |  | RP: GGCGGCCGCCAATATAGTGGAGCTCCAG | |
| Shine Dalgarno Sequence | | FP: CTATATTGGCGGCCAGAAGGAGCCACCATGGATTA | |
|  |  | RP: TAATCCATGGTGGCTCCTTCTGGCCGCCAATATAG | |
| Termination site | | FP: TGAATGGCGAATGGCGAGCCAATTTTTAAGTGTAT | |
|  |  | RP: ATACACTTAAAAATTGGCTCGCCATTCGCCATTCA | |
| -35 Sequence for pFLAG^B^-*osmY^SS-P^* | | FP: AAATTAACCCTCACTATCCCGAGCGAAAAGCTGGAGCTC | |
|  |  | RP: GAGCTCCAGCTTTTCGCTCGGGATAGTGAGGGTTAATTT | |
| Transcription Startsite for pFLAG^B^-*osmY^SS-P^* | | FP: GAGCTCCACTATATTTAACAAAAAGAAGGAGCCACCA | |
|  |  | RP: TGGTGGCTCCTTCTTTTTGTTAAATATAGTGGAGCTC | |
| **Sequencing Primer** | | | |
| T7 promoter | | TAATACGACTCACTATAG | |
| **Primer for Cloning** | | | |
| pFLAG^B^-*pcnB* | | FP: CGCGGATCCGATTTTTACCCGAGTCGCT | |
|  |  | RP: CCGGAATTCCGTCATGCGGTACCCTCAC | |
| pFLAG^B^-*osmY* | | FP : CGGGATCCGATGACTATGACAAGACTGAAGA | |
|  |  | RP: GAATTCAAGCGTCTCCTTTACCATAGT | |
| pFLAG^B^-*wzc* | | FP: CGCGGATCCGATGACAGAAAAAGTAAAACAAC | |
|  |  | RP: GAATTCTTTCGCATCCGACTTATATTCG | |
| pET-*pcnB* | | FP: CCATGCCATGGGCATTTTTACCCGAGTCGCTAA | |
|  |  | RP: CCGCTCGAGCGGTGCGGTACCCTCACGACGT | |
| **RNA Oligos** | | | |
| A_45_ oligo | | AGGGAUAGGGAUAGGGAUUAGGGAUAGGGAAAAAAAAA | |
